# Phylogenetic position and morphological polymorphism of the chafer, *Clinterocera nigra* (Coleoptera: Scarabaeidae: Cetoniinae) from Taiwan

**DOI:** 10.1080/23802359.2022.2109438

**Published:** 2022-08-22

**Authors:** Li-Wei Wu, Ming-Yu Chen, Chun-Lin Li

**Affiliations:** a Department of Life Science, Tunghai University, Taichung, Taiwan; b The Experimental Forest, National Taiwan University, Chushan, Taiwan

**Keywords:** Mitochondrial metagenomics, chafer, mitochondrion, melanistic, myrmecophilous

## Abstract

Three mitochondrial genomes of the cetoniine beetle, *Clinterocera nigra* (Kano, 1931) were assembled via next-generation sequencing. The newly sequenced mitogenomes all have 37 genes, showing standard gene order and annotation as the other insects. To examine their phylogenetic positions and relationships between their elytral color (red-spot and melanistic forms) and sequence variation, a total of 118 public mitogenomes of Scarabaeidae were used to infer a maximum-likelihood (ML) tree. Our results show that the melanistic form is grouped within red-spot ones, revealing a population level variation on the elytra color. Our work also provides the first mitogenomic reference of myrmecophilous chafers.

The myrmecophilous genus, *Clinterocera* Motschulsky, 1858 (Coleoptera: Scarabaeidae: Cetoniinae: Cremastocheilini), is distributed in Southeast Asia, consisting of 29 species (Xu et al. 2018). Their diagnostic features have been distinctly characterized by reduced tarsomeres, triangularly enlarged antennal scape and cup-shaped prementum which might be adapted to symbiosis with ants (Qiu and Xu 2016). Kano (1926) first reported *Callinomes davidis* Fairmaire, 1878 from Taiwan, and it was lately transferred to *Clinterocera* by Medvedev (1964). The species can be easily recognized by well-developed red spots on elytra. However, there was a subspecies, *Cl. nigra*, named by Kano (1931) with the elytral red spots largely reduced (Figure 1). This taxonomic arrangement was followed and has never been challenged (i.e. Sakai and Nagai 1998; Smetana 2006; Bezděk 2016) until Xu et al. (2018) restricted the distribution of *Cl. davidis* in mainland China and raised *Cl. d. nigra* to specific rank. We herein conducted a mitogenomic comparison with three *Clinterocera* specimens in different color form from Taiwan.

**Figure 1.**
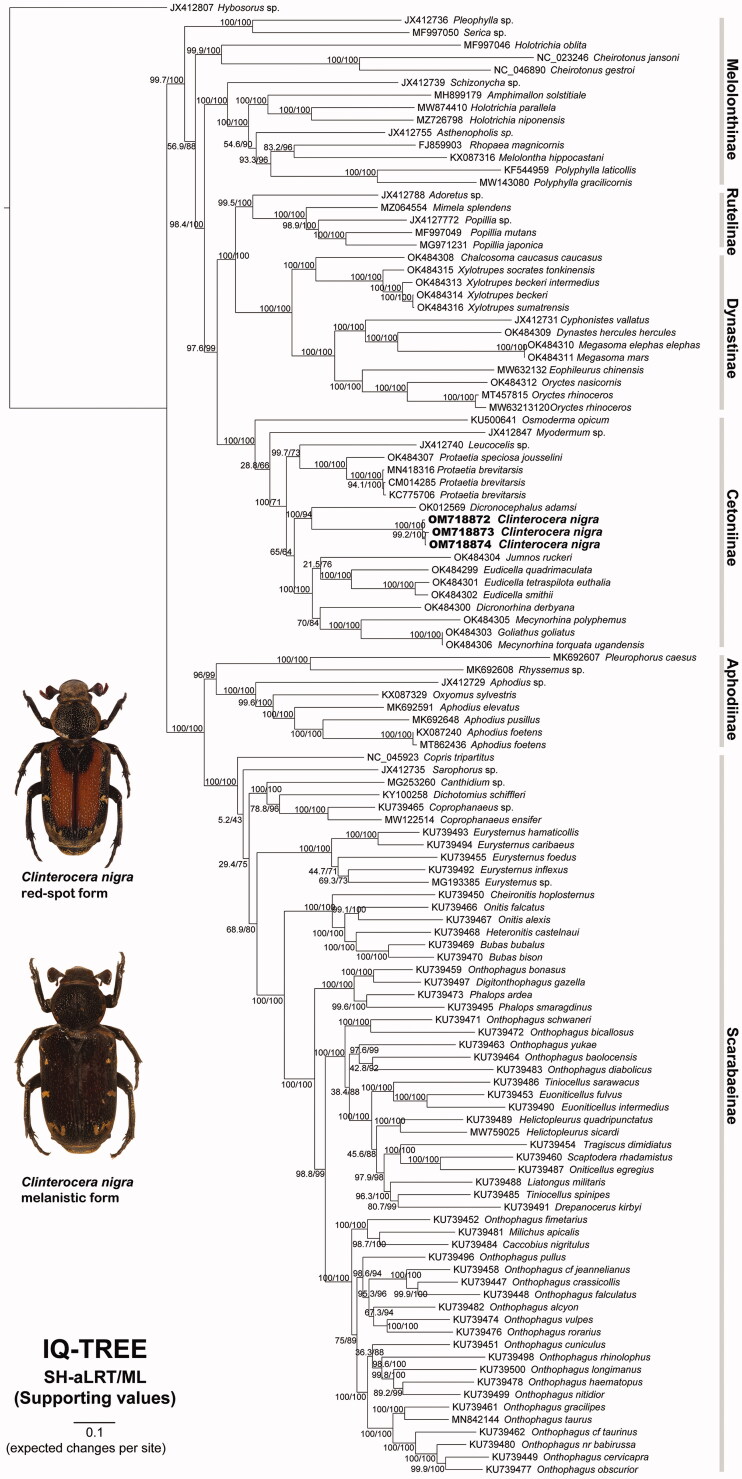
The ML phylogeny of the Scarabaeidae based on 118 mitogenomes using IQ-TREE. The dorsal view of red-spot and melanistic forms of *Clinterocera nigra* is shown. Nodal supports of SH-aLRT/ML: SH-like approximate likelihood ratio test (Guindon et al. 2010)/ML ultrafast bootstrapping (Minh et al. 2013).

For evaluating mitogenomic variation, three specimens were collected in this work: two red elytral spots specimens of *Cl. nigra* were collected from Taipei (coordinate: N: 24.9928, E: 121.5484; DNA code: 20CL07001) and Xitou, Nantou (N: 23.6738, E: 120.7969; DNA code: 20CL07002); the melanistic specimen was collected at its type locality (Neimoupu, Nantou; N: 23.6898, E: 120.8505; DNA code: 20CL07003). Genomic DNAs were extracted using Gentra Puregene Tissue Kit (Gentra Systems, Minneapolis, MN) and all the extracts were fragmented into 200–300 bp for next-generation sequencing via Illumina Novaseq 6000 platform (San Diego, CA).

Each NGS dataset was trimmed out low-quality region (<Q20) and then *de novo* assembled the remaining with 97% similarity using CLC Genom.ics Workbench (CLC Bio, Aarhus, Denmark) and megahit 1.2 (Li et al. 2015). The reference library (Cheng et al. 2021) was used to filter out mitogenome-like sequence reads (threshold set to 70% similarity). The obtained reads were then combined, checked, and corrected using Sequencher 4.10 (GeneCode, Boston, MA). Three mitogenomic sequences were obtained: 17,014 bp for 20CL07001 (average coverage = 10,038–19,844); 17,421 bp for 20CL07002 (average coverage = 7–272); 17,709 bp for 20CL07003 (average coverage = 6567). Gene regions and annotation were predicted with MITOS (Donath et al. 2019), and these three newly sequenced mitogenomes all have standard 37 mitochondrial genes, presenting standard gene order and direction as other insects (Boore 1999).

*Cl. nigra*, a total of 118 mitogenomes, comprising three our samples and 115 public scarabaeids, were used to infer their phylogenetic relationships. Sequences were separately aligned based on gene region using MUSCLE (Edgar 2004), implied in MEGA-X (Kumar et al. 2018). All the aligned genes were concatenated (15,521 bp in length) into a sequence matrix, and manually formatted the matrix as phylip or nexus formats for downstream analysis. The *Hybosorus* sp. (Hybosoridae) was set to outgroup and 16 partitions (13 protein-coding genes, *rrnL*, *rrnS*, and tRNAs) with GTR + G model were set. The maximum-likelihood (ML) phylogeny was reconstructed using IQ-TREE (Nguyen et al. 2015), and the nodal supports were evaluated by 1000 replicates of bootstrapping and the SH-like approximate likelihood ratio test (SH-aLRT) (Guindon et al. 2010).

The phylogenetic relationships show that three *Cl. nigra* individuals are strongly grouped in a clade, sister to *Dicronocephalus* under the tribe Cremastocheilini (Figure 1). The result is consistent with the previous study (Šípek et al. 2016). Meanwhile, the melanistic *Cl. nigra* (20CL07003) are grouped within the other two red-spot samples (Figure 1), even genetic distance between melanistic and red-spot forms (K2P genetic distance: 0.3% and 0.9%) is smaller than the distances between two red-spot samples (K2P genetic distance: 1.2%), supporting the taxonomic treatment by Xu et al. (2018).

## Data Availability

The data that support the findings of this study are available in GenBank at https://www.ncbi.nlm.nih.gov/genbank/, reference numbers: OM718872–OM718874. The raw sequence data were deposited in SRA database under BioProject (PRJNA806699). SRA and BioSample are 20CL07001 (SRS11990152, SAMN25894942), 20CL07002 (SRS11990153, SAMN25894943), and 20CL07003 (SRS11990154, SAMN25894944), respectively. Specimens of *Clinterocera nigra* were deposited at National Museum of Natural Science, Taichung (contact person: Jing-Fu Tsai, email: jftsai.nmns@gmail.com) under the vouchers of NMMS ENT 8515-1 (20CL07001), NMMS ENT 8515-2 (20CL07002), and NMNS 8515-3 (20CL07003), respectively.
